# Type 2 Diabetic Sepsis Patients Have a Lower Mortality Rate in Pioglitazone Use: A Nationwide 15-Year Propensity Score Matching Observational Study in Taiwan

**DOI:** 10.1155/2021/4916777

**Published:** 2021-07-23

**Authors:** Ming-Shun Hsieh, Sung-Yuan Hu, Shu-Hui Liao, Chia-Ming Chang, Vivian Chia-Rong Hsieh, Chorng-Kuang How

**Affiliations:** ^1^Department of Emergency Medicine, Taipei Veterans General Hospital, Taoyuan Branch, Taoyuan, Taiwan; ^2^Department of Emergency Medicine, Taipei Veterans General Hospital, Taipei, Taiwan; ^3^School of Medicine, National Yang Ming Chiao Tung University, Taipei, Taiwan; ^4^Department of Emergency Medicine, Taichung Veterans General Hospital, Taichung, Taiwan; ^5^Department of Pathology and Laboratory, Taipei Veterans General Hospital, Taoyuan Branch, Taoyuan, Taiwan; ^6^Department of Health Services Administration, China Medical University, Taichung, Taiwan; ^7^Kinmen Hospital, Ministry of Health and Welfare, Kinmen, Taiwan

## Abstract

**Background:**

Pioglitazone use via the PPAR*γ* agonist in sepsis patients is inconclusive. It was based on a great number of animal studies. However, except for information from animal studies, there are merely any data of human studies for reference.

**Methods:**

This study was conducted by a unique database including 1.6 million diabetic patients. From 1999 to 2013, a total of 145,327 type 2 diabetic patients, first admitted for sepsis, were enrolled. Propensity score matching was conducted in a 1 : 5 ratio between pioglitazone users and nonusers. Multivariate logistic regression was conducted to evaluate the adjusted odds ratios (aORs) of hospital mortality in pioglitazone users. Further stratification analysis was done and Kaplan–Meier plot was used.

**Results:**

A total of 9,310 sepsis pioglitazone users (defined as “ever” use of pioglitazone in any dose within 3 months prior to the first admission for sepsis) and 46,550 matched nonusers were retrieved, respectively. In the multivariate logistic regression model, the cohort of pioglitazone users (9,310) had a decreased aOR of 0.95 (95% CI, 0.89–1.02) of sepsis mortality. Further stratification analysis demonstrated that “chronic pioglitazone users” (defined as “at least” 4-week drug use within 3 months) (3,399) were more associated with significant aOR of 0.80 (95% CI, 0.72–0.89) in reducing sepsis mortality.

**Conclusions:**

This first human cohort study demonstrated the potential protective effect of chronic pioglitazone use in type 2 diabetic sepsis patients.

## 1. Introduction

Sepsis is a major cause of mortality worldwide, especially in the immunocompromised patients, such as those with multiple comorbidities [1–3]. Sepsis is a complex syndrome that is induced by severe infection with a series of unregulated immune responses, caused majorly by the proinflammatory cytokines. Acute organ failure and subsequent high mortality rate will induce long-term morbidities, such as stroke and cardiovascular diseases [3, 4]. Despite advances in treatment strategies, therapies to mitigate the severity of sepsis are currently unsatisfactory [5].

Thiazolidinediones (TZD), a kind of oral antidiabetic drugs (OADs), are used for the treatment of type 2 diabetes via being the insulin sensitizers [6]. Currently, pioglitazone is the only TZD available in the market. It is proposed to have a protection effect during the sepsis course by acting as the peroxisome proliferator-activated receptor-gamma (PPAR*γ*) agonist [7]. Because of multiple concerns about TZDs' complications, for example, cardiovascular disease and urinary bladder cancer, the prescriptions of TZD decreased significantly, from 9.20% in 2006 to 2.86% in 2012 in Taiwan [8–10].

Accumulating evidence in animal studies demonstrated that PPAR*γ* agonists improved the outcomes of sepsis via multiple mechanisms [11]. In the mouse model, pioglitazone administration decreased inflammation and improved survival of sepsis induced by cecal ligation and puncture (CLP) [12]. Because of the growing amount of evidence, the randomized clinical trial of pioglitazone use in sepsis patients is underway [13]. However, currently, there are limited data on this topic in humans, especially in type 2 diabetic patients, because the varied levels of diabetic complication burdens in each person were not easily compared [14].

In the current study, we used a specially applied nationwide database of diabetic patients, from 1999 to 2013, with the first admission for sepsis to evaluate the impacts of pioglitazone use in sepsis with the main outcome of the total hospital mortality.

This cohort study addressed the selection bias from diabetic severity by using the propensity score matching and simulated a real-world clinical trial to compare subjects in each group.

## 2. Methods

### 2.1. Data Sources and Study Participants

We conducted this cohort study by using the National Health Insurance Research Database (NHIRD) of Taiwan. The National Health Insurance program in Taiwan currently provides coverage for more than 99% of the entire population. The National Health Insurance in Taiwan provides excellent healthcare service to the people [15–18]. The deidentified patient information and claims data were released to the National Health Research Institute to establish the NHIRD. The diagnosis codes of the International Classification of Diseases, Ninth Revision, Clinical Modification (ICD-9-CM) are used.

From the NHIRD, we conducted this study by using the specially applied database of “Longitudinal Cohort of Diabetes Patients (LHDB)” which enrolled a longitudinal cohort of 1.6 million newly diagnosed diabetic patients from 1999 to 2013. We retrieved data from LHDB to constitute the study and comparison cohorts, composed by type 2 diabetic patients with a first admission for sepsis with and without pioglitazone use.

### 2.2. Definition of Sepsis and Baseline Comorbidities

The diagnosis of sepsis in the current study was retrieved using the ICD-9-CM code 038 plus a main infection diagnosis with antibiotics prescription. The accuracy of sepsis diagnosis in the NHIRD has been validated [19]. The patients were defined as having certain comorbidities if they had at least 2 outpatient service claims or if they had a single hospitalization in which the certain comorbidities were found. The index date was defined as the first admission date for sepsis.

### 2.3. Definition of Drug Use in Pioglitazone

In this study, if a patient received the prescription of any dose of pioglitazone within 3 months prior to the index admission for sepsis, he or she would be defined as a pioglitazone user or “ever use” pioglitazone. Throughout the whole study, we used the above definition to describe any drug use.

To reduce the medical expenditure for the stable patients of type 2 diabetes or other chronic diseases, the physicians can use the refill card of consecutive prescription for 3 months rather than prescribing the drugs week by week or only 3 days. Based on the above medical regulation and culture in Taiwan, we therefore defined a person as a “chronic” pioglitazone user if he or she was prescribed pioglitazone for at least 4 weeks within 3 months prior to the first admission for sepsis [20].

### 2.4. Propensity Score Matching

Propensity score matching could reduce the selection bias because it allowed the bundling of many confounding factors which were frequently presented in the observation studies [21–23]. We calculated the propensity score using the multivariate logistic regression by entering the baseline covariates which included age, sex, comorbidities, insurance premium, and complication severity of type 2 DM.

Since type 2 diabetes related complications may be the most important factor to determine the hospital outcome, we matched 1 study cohort patient with 5 comparison cohort patients according to propensity score and obtained a dataset composed of matched patients who had a statistically identical likelihood of severity of diabetic complications.

In the database, the individual insurance premium fee paid was a useful surrogate for the household income level [24].

### 2.5. Selection Process

**Figure 1:**
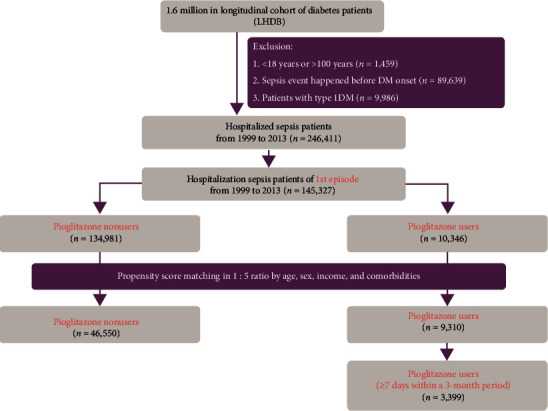
The participant selection process in the study and comparison cohorts.

The algorithm used for participant selection for the study and comparison cohorts is shown in Figure 1. Patients aged <18 or >100 years, patients with type 1 diabetes, and patients infected with human immunodeficiency virus were excluded from this study. Since the database contains deidentified data for research, our study was exempted from the requirement of informed consent from participants. This study was approved by the Institutional Review Board of Taipei Veterans General Hospital (2020-01-012CC) and China Medical University (CMUH104-REC2-115).

### 2.6. Comparison between the Study and Matched Cohorts

Differences in demographic characteristics, insurance premium, baseline comorbidities, medications (including nonsteroidal anti-inflammatory drugs (NSAIDs), aspirin, statins, biguanides, dipeptidyl peptidase-4 inhibitors (DPP-4 inhibitors), sulfonylureas, pioglitazone, insulin, immunosuppressants, and steroids), infection sites, adapted diabetes complications severity index (aDCSI) score which was a representation of diabetes complication severity, length of hospital stay, and the total hospital mortality were examined using the chi-squared test and two-sample *t*-test.

### 2.7. Logistic Regression and Kaplan–Meier Analysis

Odds ratios (ORs) and 95% confidence intervals (95% CIs) were calculated for each variable in the logistic regression model. Adjusted ORs (aORs) for the total hospital mortality were obtained after adjusting for potential confounders, including age, sex, income, and comorbidities, in the multivariate logistic regression analysis. Kaplan–Meier analysis with log-rank test was conducted to compare the difference in the outcomes of total hospital mortality between the study and comparison cohorts (i.e., pioglitazone users versus nonusers). The statistical analyses were performed using the SAS 9.4 statistical package (SAS Institute, Inc., Cary, NC, USA). A *P* value of 0.05 was considered significant.

## 3. Results

### 3.1. Demographic Characteristics and Baseline Comorbidities between the Enrolled Sepsis Patients of Pioglitazone Users and Nonusers

From the LHDB, we initially retrieved a total of 145,327 type 2 diabetic patients with the first admission for sepsis from 1999 to 2013. After propensity score matching, a total of 9,310 pioglitazone users and 46,550 nonusers were included for further analysis.

Before PS matching, the mean ages of pioglitazone users and nonusers were 67.08 ± 12.62 and 71.13 ± 13.90 years, respectively. After PS matching, the mean ages of pioglitazone users and nonusers were 68.11 ± 12.50 and 68.96 ± 13.18 years, respectively. The detailed demographic characteristics are shown in Table 1. Before matching, a greater proportion of pioglitazone users than nonusers received treatment with statins (30.85% versus 11.52%), biguanide (60.48% versus 28.80%), DPP-4 inhibitors (21.35% versus 3.52%), sulfonylurea (65.17% versus 31.84%), and insulin (68.11% versus 43.37%) (all *P* < 0.001). The pioglitazone users had fewer respiratory system infection sites compared to nonusers (46.24% versus 51.76%) (*P* < 0.001). The pioglitazone users had more severe complication burdens (aDCSI score ≥ 5, 16.30% of pioglitazone users versus 8.95% of nonusers) (*P* < 0.001). However, the pioglitazone users had lower total hospital mortality rate (15.83% versus 18.6%) and shorter length of hospital stay (mean, 11 versus 12 days) than the nonusers.

### 3.2. Regression Model of the Total Hospital Mortality

In the logistic regression model, after further adjusting for age, sex, income, and comorbidities, the pioglitazone users (ever use) were shown to have a nonsignificant aOR slightly less than unity for total hospital mortality (aOR = 0.95 (95% CI, 0.89–1.03)) (Table 2).

In the further stratification analysis, the patients who were classified as “chronic pioglitazone users” demonstrated the significant aOR for total hospital mortality (aOR = 0.80 (95% CI, 0.72–0.89), *P* < 0.05) (Table 3).

### 3.3. Kaplan–Meier Analysis of the Total Hospital Mortality

**Figure 2:**
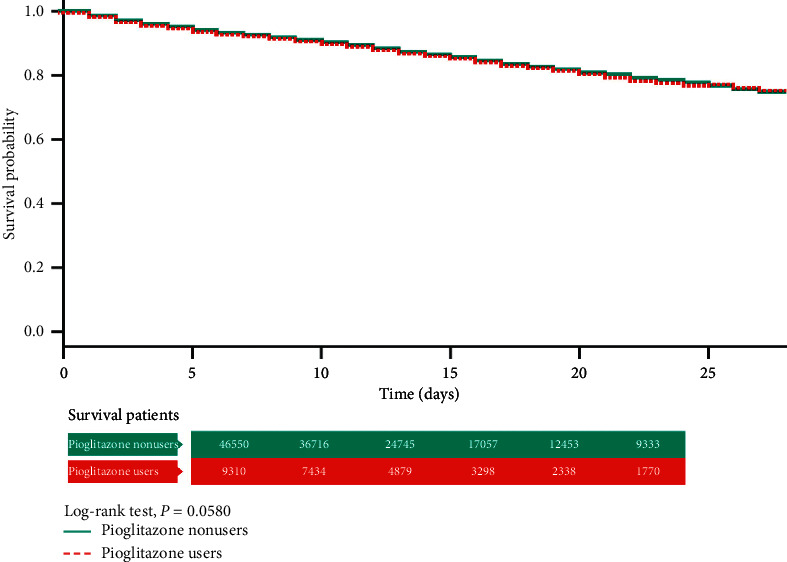
Kaplan–Meier analysis with log-rank test of the total hospital mortality in pioglitazone users (in any dose within 3 months prior to the first admission for sepsis) and nonusers.

**Figure 3:**
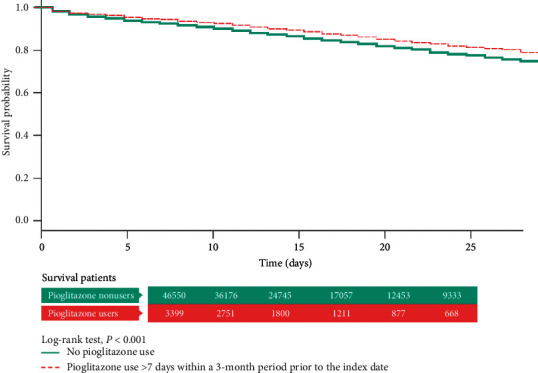
Kaplan–Meier analysis with log-rank test of the total hospital mortality in pioglitazone users (at least 4 weeks' drug use within 3 months) and nonusers.

In the Kaplan–Meier analysis with log-rank test, the total hospital mortality did not differ significantly between the pioglitazone users (“ever use”) and nonusers (Figure 2). However, in the “chronic pioglitazone users,” it was obvious that the cumulative survival rate was much better than that in the nonusers (*P* < 0.01) (Figure 3).

## 4. Discussion

In this real-world study, by using the nationwide database of diabetic patients with propensity score matching, we demonstrated that pioglitazone use can exert a significantly protective effect in “chronic pioglitazone users.” This finding has been proved in multiple animal studies for a long term. However, it remained controversial in human beings. To the best of our knowledge, this is the first and largest cohort study of type 2 diabetic sepsis patients that simulated the human clinical trial via propensity score matching to examine the protective effect of pioglitazone. Our finding will surely attract more and more attention focusing on the potential of pioglitazone in sepsis.

PPARs encoded by separate genes, PPAR*α*, PPAR*β⁄δ*, and PPAR*γ*, are expressed by a variety of cells of the immune system including macrophages, *B* and *T*, and monocytes, lymphocyte, natural killer cells, dendritic cells, mass cells, neutrophils, and eosinophils [25]. PPARs have received attention till now, since they play pivotal regulators in adipocyte differentiation, glucose homeostasis, and immune regulation. In the current study, we focus on the role of immune modification of pioglitazone which is a kind of TZDs, activating as the PPAR*γ* agonist.

PPAR*γ* agonists can be simply classified into natural and artificial ones, respectively. Natural PPAR*γ* agonists include saturated and unsaturated fatty acids, eicosanoid derivatives, such as 15-deoxy-Δ^12,14^-prostaglandin J2 (15d-PGJ2), and oleic and nitrated linoleic acids. Synthetic PPAR*γ* agonists are represented by TZDs, such as pioglitazone, rosiglitazone, troglitazone, and ciglitazone. TZDs (agonist) function via activating the PPAR*γ* receptor [26, 27]. In the absence of PPAR*γ* agonists, these ligands remain inactive via binding to the corepressors.

Pioglitazone is currently the only available TZD in the market, since rosiglitazone has been suspended in Taiwan in 2011 due to its potentially increased risk of myocardial infarction and decompensated heart failure [28]. Pioglitazone targets the transcription of PPAR*γ* and is involved in metabolic homeostasis, and, most important of all, it much improves insulin sensitivity. This mechanism provides a choice to type 2 diabetic patients before receiving insulin injection therapy. Following the suspension of rosiglitazone in 2011, pioglitazone later became another target of criticism, including that pioglitazone (1) increased the risk of osteoporosis and (2) increased the risk of urinary bladder cancer. However, supporting data to pioglitazone passed these [29, 30]. Moreover, many studies and specialist opinion support that pioglitazone should continue to be used in T2D treatment [31–33].

In addition to helping glucose control, the activation of PPAR*γ* agonist of pioglitazone contributes to the modulation of inflammation [34]. Pioglitazone improving bacterial elimination in the peripheral blood, via inhibition of proinflammatory molecules such as IL-6, TNF, IL-1, and IL-12, has been well documented. It also enhanced bacterial elimination in the liver by increasing the phagocytic and bactericidal activities [11]. Furthermore, accumulated evidence of animal models demonstrated that pioglitazone is effective in the prevention and treatment of sepsis in mice cecal ligation and puncture (CLP) model [35–37]. In the type 2 diabetic patients, who are prone to infection diseases, TZDs use with pioglitazone should be an adequate choice via the anti-inflammatory and enhancing bactericidal effects. Our study demonstrated the protective effect in sepsis patients, which added a new important evidence in human body.

The repeated or accumulated doses of pioglitazone presented the dose-effect relationship compared with the single-dose use as observed in this study. Besides, PPAR*γ* agonists are known to upregulate their receptors' expression, which render the greater anticipated effects of repeated or accumulating dosing [38]. However, currently, which is the optimal dose and which stage to start pioglitazone treatment in sepsis deserve further investigation. Combining with other studies, we inferred that the initial or chronic use of pioglitazone might inhibit the cytokine storm and therefore reduced the acute organ failure in the first fulminant stage. Continuous use during the sepsis course remains for further study.

### 4.1. Limitations

This study has several limitations. First, this study lacked certain important laboratory data, that is, initial blood glucose level and hemoglobin A1C (HbA1c), which was an inevitable weak point in administrative database studies. However, we had demonstrated that there was no association between initial blood glucose, HbA1c, and hospital outcomes of sepsis in our previous hospital-based study; the lack of initial blood glucose level and HbA1c may be not as important as previously thought [14]. Second, the impact of pioglitazone use on every sepsis stage remains further examined since systemic inflammatory response syndrome (SIRS) and subsequent compensatory anti-inflammatory response syndrome (CARS) might occur in sequence or concurrently, named as mixed antagonist response syndrome (MARS). Our study design mainly focused on the first stage, that is, SIRS.

## 5. Conclusion

In this study, we demonstrated that, currently, regular preadmission pioglitazone use improved the total hospital mortality in type 2 diabetic sepsis patients after considering multiple variables, including comorbidities and household income.

## Figures and Tables

**Table 1:** Demographic characteristics, comorbidities, and diabetic complications of type 2 diabetic sepsis patients between pioglitazone users and nonusers.

Variables	Before matching						After PS matching					
	Nonusers (*n* = 134,981)		Users (*n* = 10,346)		*P*^*∗*^ value	Standardized mean difference	Nonusers (*n* = 46,550)		Users (*n* = 9,310)		*P*^*∗*^ value	Standardized mean difference
	*n*	%	*n*	%			*n*	%	*n*	%		
*Sex*					<0.001						0.361	0.010
Female	61,623	45.65	5,018	48.50		0.057	22,289	47.88	4,506	48.40		
Male	73,358	54.35	5,328	51.50		0.057	24,261	52.12	4,804	51.60		

*Age, years*					<0.001						0.004	
18–29	600	0.44	12	0.12		0.062	116	0.25	9	0.10		0.037
30–49	11,323	8.39	994	9.61		0.043	4,079	8.76	766	8.23		0.019
50–69	42,086	31.18	4,718	45.60		0.300	18,941	40.69	3,969	42.63		0.039
70–89	74,625	55.29	4,468	43.19		0.244	22,587	48.52	4,412	47.39		0.023
90–100	6,347	4.70	154	1.49		0.186	827	1.78	154	1.65		0.009
Mean (SD)	71.13 (13.90)		67.08 (12.62)		<0.001	0.305	68.96 (13.18)		68.11 (12.50)		<0.001	0.066

*Insurance premium (NT dollars)*											0.395	
<20000	82,771	61.32	4,705	45.48		0.322	22,918	49.23	4,523	48.58		0.013
20000–40000	43,501	32.23	4,575	44.22		0.249	19,836	42.61	4,023	43.21		0.012
40000–60000	6,461	4.79	800	7.73		0.122	2,890	6.21	598	6.42		0.009
>60000	2,248	1.67	266	2.57		0.063	906	1.95	166	1.78		0.012

*Comorbidity*												
HTN	101,906	75.50	8,732	84.4	<0.001	0.224	39,091	83.98	7,728	83.01	0.020	0.026
Hyperlipidemia	62,096	46.00	7,397	71.5	<0.001	0.536	31,910	68.55	6,363	68.35	0.698	0.004
COPD	63,740	47.22	4,049	39.14	<0.001	0.164	19,357	41.58	3,868	41.55	0.948	0.001
CLD	45,715	33.87	4,091	39.54	<0.001	0.118	18,691	40.15	3,674	39.46	0.215	0.014
CKD	61,015	45.20	5,988	57.88	<0.001	0.256	25,183	54.10	5,093	54.70	0.284	0.012
PAOD	20,462	15.16	1,832	17.71	<0.001	0.069	7,625	16.38	1,597	17.15	0.066	0.021
IHD	64,874	48.06	5,144	49.72	0.011	0.033	23,337	50.13	4,668	50.14	0.990	0
Stroke	60,780	45.03	4,343	41.98	<0.001	0.062	19,974	42.91	4,063	43.64	0.192	0.015
Cancer	38,145	28.26	2,693	26.03	<0.001	0.050	12,649	27.17	2,541	27.29	0.811	0.003

*Drug use*												
NSAID	74,050	54.86	5,534	53.49	0.070	0.028	26,508	56.95	4,968	53.36	<0.001	0.072
Aspirin	15,122	11.20	844	8.16	<0.001	0.103	5,201	11.17	759	8.15	<0.001	0.102
Statins	15,549	11.52	3,192	30.85	<0.001	0.487	7,585	16.29	2,763	29.68	<0.001	0.322
Biguanides	38,874	28.80	6,257	60.48	<0.001	0.672	14,986	32.19	5,667	60.87	<0.001	0.600
DPP-4 inhibitors	4,751	3.52	2,209	21.35	<0.001	0.561	2,145	4.61	1,932	20.75	<0.001	0.500
Sulfonylureas	42,982	31.84	6,743	65.17	<0.001	0.707	16,255	34.92	6,098	65.50	<0.001	0.642
Pioglitazone	0	0	3,764	36.38	—	—	0	0	3,399	36.51	<0.001	1.072
Insulin	58,539	43.37	7,047	68.11	<0.001	0.514	20,724	44.52	6,296	67.63	<.0001	0.479
Immunosuppressants	475	0.35	57	0.55	0.001	0.030	224	0.48	41	0.44	0.600	0.006
Steroids	38,618	28.61	2,876	27.8	0.078	0.018	13,213	28.38	2,608	28.01	0.467	0.008

*Infection site*												
Central nervous	1,411	1.05	100	0.97	0.446	0.008	525	1.13	88	0.95	0.122	0.018
Respiratory	69,870	51.76	4,784	46.24	<0.001	0.111	21,986	47.23	4,416	47.43	0.721	0.004
Cardiovascular	1,938	1.44	156	1.51	0.553	0.006	656	1.41	133	1.43	0.885	0.002
Gastrointestinal	20,361	15.08	1,795	17.35	<0.001	0.061	7,459	16.02	1,596	17.14	0.007	0.030
Genitourinary	69,360	51.39	5,472	52.89	0.003	0.030	23,449	50.37	4,959	53.27	<.001	0.058
Soft tissue/bone	24,980	18.51	2,262	21.86	<0.001	0.084	8,782	18.87	1,991	21.39	<.001	0.063
Device-related	4,847	3.59	431	4.17	0.002	0.030	1,946	4.18	354	3.80	0.093	0.019
Others	34,048	25.22	2,568	24.82	0.362	0.009	10,969	23.56	2,309	24.8	0.105	0.029

*aDCSI score*					<0.001						<0.001	
0	38,882	28.81	2,092	20.22		0.201	12,732	27.35	1,937	20.81		0.154
1	15,563	11.53	1,485	14.35		0.084	5,818	12.50	1,319	14.17		0.049
2	36,729	27.21	2,288	22.11		0.118	12,078	25.95	2,067	22.20		0.088
3	13,194	9.77	1,379	13.33		0.111	4,844	10.41	1,243	13.35		0.091
4	18,532	13.73	1,416	13.69		0.001	6,425	13.80	1,281	13.76		0.001
≥5	12,081	8.95	1,686	16.30		0.223	4,653	10.00	1,463	15.71		0.171
Length of hospital stay (days) (median)	12		11		<0.001	0.056	11		11		0.0215	0.027
Total hospital mortality	25,102	18.6	1,638	15.83		0.073	7,947	17.07	1,523	16.36	0.0941	0.019

^*∗*^Chi-square test.

**Table 2:** Logistic regression model to estimate the OR and 95% CI of the total hospital mortality in pioglitazone users and nonusers.

Variable	Outcome = total hospital mortality	
	Crude OR	Adjusted OR
	(95% CI)	(95% CI)
Pioglitazone use	0.95(0.88–1.01)	0.95(0.89–1.03)

*Sex*		
Female	1 (ref)	1 (ref)
Male	1.59 (1.52–1.66)^*∗*^	1.54 (1.47–1.62)^*∗*^

*Age, years*		
18–29 years	1 (ref)	1 (ref)
30–49 years	1.45 (0.78–2.70)	1.39 (0.74–2.63)
50–69 years	1.94 (1.04–3.60)^*∗*^	1.67 (0.89–3.13)^*∗*^
70–89 years	2.36 (1.27–4.39)^*∗*^	1.93 (1.03–3.62)^*∗*^
90–100 years	3.34 (1.77–6.30)^*∗*^	2.85 (1.49–5.45)^*∗*^

*Insurance premium (NT dollars)*		
<20000	1 (ref)	1 (ref)
20000–40000	0.60 (0.58–0.63)^*∗*^	0.61 (0.58–0.64)^*∗*^
40000–60000	0.66 (0.60–0.73)^*∗*^	0.64 (0.58–0.71)^*∗*^
>60000	0.92 (0.79–1.08)	0.76 (0.65–0.90)^*∗*^

*Baseline comorbidity (Ref* *=* *Non-)*		
HTN	1.00 (0.94–1.06)	0.95 (0.88–1.01)
Hyperlipidemia	0.77 (0.73–0.80)^*∗*^	0.77 (0.74–0.81)^*∗*^
COPD	0.96 (0.92–1.01)	0.84 (0.80–0.88)^*∗*^
CLD	1.14 (1.09–1.19)^*∗*^	1.06 (1.01–1.11)^*∗*^
CKD	1.38 (1.32–1.45)^*∗*^	1.35 (1.29–1.42)^*∗*^
PAOD	1.16 (1.09–1.22)^*∗*^	1.11 (1.05–1.18)^*∗*^
IHD	1.02 (0.97–1.06)	0.99 (0.95–1.04)
Stroke	0.99 (0.94–1.03)	0.96 (0.91–1.00)
Cancer	3.29 (3.14–3.44)^*∗*^	3.23 (3.08–3.39)^*∗*^

Adjusted OR: adjusted for age, sex, insurance premium, and comorbidities in logistic regression. Any dose of pioglitazone within 3 months prior to the index admission for sepsis; ^*∗*^*P* < 0.05. CI, confidence interval; CKD, chronic kidney disease; CLD, chronic liver disease; COPD, chronic obstructive pulmonary disease; DPP-4 inhibitor, dipeptidyl peptidase-4 inhibitor; HTN, hypertension; IHD, ischemic heart disease; NSAID, nonsteroidal anti-inflammatory drug; NT, new Taiwan; OR, odds ratio; PAOD, peripheral arterial occlusion disease.

**Table 3:** Logistic regression model to estimate the OR and 95% CI of total hospital mortality in pioglitazone users (≥7 days within 3 months) and nonusers.

Variable	Outcome = total hospital mortality	
	Crude OR	Adjusted OR
	(95% CI)	(95% CI)
Pioglitazone	0.76 (0.69–0.84)^*∗*^	0.80 (0.72–0.89)^*∗*^

*Sex*		
Female	1 (reference)	1 (reference)
Male	1.59 (1.52–1.66)^*∗*^	1.54 (1.47–1.62)
*Age, years*		
18–29 years	1 (ref)	1 (ref)
30–49 years	1.45 (0.78–2.70)	1.40 (0.74–2.64)
50–69 years	1.94 (1.04–3.60)^*∗*^	1.67 (0.89–3.14)
70–89 years	2.36 (1.27–4.39)^*∗*^	1.94 (1.03–3.64)
90–100 years	3.34 (1.77–6.30)^*∗*^	2.85 (1.49–5.46)

*Insurance premium (NT dollars)*		
<20000	1 (ref)	1 (ref)
20000–40000	0.60 (0.58–0.63)^*∗*^	0.61 (0.58–0.64)
40000–60000	0.66 (0.60–0.73)^*∗*^	0.64 (0.58–0.71)
>60000	0.92 (0.79–1.08)	0.76 (0.65–0.90)

*Comorbidity (Ref* *=* *Non-)*		
HTN	1.00 (0.94–1.06)	0.95 (0.88–1.01)
Hyperlipidemia	0.77 (0.73–0.80)^*∗*^	0.77 (0.74–0.81)
COPD	0.96 (0.92–1.01)	0.84 (0.80–0.88)
CLD	1.14 (1.09–1.19)^*∗*^	1.06 (1.01–1.11)
CKD	1.38 (1.32–1.45)^*∗*^	1.35 (1.29–1.42)
PAOD	1.16 (1.09–1.22)^*∗*^	1.11 (1.05–1.18)
IHD	1.02 (0.97–1.06)	0.99 (0.94–1.04)
Stroke	0.99 (0.94–1.03)	0.96 (0.91–1.00)
Cancer	3.29 (3.14–3.44)^*∗*^	3.23 (3.08–3.38)

Adjusted OR: adjusted for age, sex, insurance premium, and comorbidities in logistic regression. ^*∗*^*P* < 0.05; ≥7 days within 3 months prior to the index admission for sepsis. CI, confidence interval; CKD, chronic kidney disease; CLD, chronic liver disease; COPD, chronic obstructive pulmonary disease; DPP-4 inhibitor, dipeptidyl peptidase-4 inhibitor; HTN, hypertension; IHD, ischemic heart disease; NSAID, nonsteroidal anti-inflammatory drug; NT, new Taiwan; OR, odds ratio; PAOD, peripheral arterial occlusion disease.

## Data Availability

The data that support the findings of this study were taken from the LHDB, but restrictions apply to the availability of these data, which were used under license for the current study. So the data are not publicly available. The data are however available from the authors upon reasonable request.

## Additional Points

*Highlights*. (1) This nationwide database in Taiwan which included 1.6 million diabetic patients from 1999 to 2013 was used to examine the potential protective effect of pioglitazone in type 2 diabetic sepsis patients. (2) Propensity score matching to avoid selection bias and aDCSI score (representation of DM severity) to evaluate diabetes severity were addressed to retrieve the pioglitazone users (9,310) and nonusers (46,550) as the study and comparison cohort. (3) To the best of our knowledge, this is the first and largest cohort study to examine the potentially protective effects from pioglitazone use in sepsis, which was limited to animal studies. (4) In the stratification analysis, the study results demonstrated that pioglitazone use (at least 7 days within 3 months) was significantly associated with decreased mortality in sepsis patients.

## Supplementary Materials

Supplementary Materials Supplementary Table 1: infection site classification by ICD-9-CM and ICD-10-CM.
