# Supplementary material for: Type 2 Diabetic Sepsis Patients Have a Lower Mortality Rate in Pioglitazone Use: A Nationwide 15-Year Propensity Score Matching Observational Study in Taiwan
Source: Emerg Med Int. 2021 Jul 23;2021:4916777. doi: 10.1155/2021/4916777 (PMC8363455; doi:10.1155/2021/4916777)
Supplement: Supplementary Materials — Supplementary Table 1: infection site classification by ICD-9-CM and ICD-10-CM. [file 4916777.f1.docx]

**Supplementary table 1**

**Infection site classification by ICD-9-CM and ICD-10-CM**

| **Diagnostic category** | **ICD-9-CM** | **ICD-10-CM** |
| --- | --- | --- |
| 1. **Central nervous system infection** | | |
| Bacterial meningitis | 320 | G00 |
| Meningitis, unspecified | 322 | G03.0,G03.1,G03.8,G03.9 |
| Central nervous system abscess | 324 | G06,G07 |
| Phlebitis of intracranial sinus | 325 | G08 |
| 1. **Respiratory system infection** | | |
| Acute sinusitis | 461 | J01 |
| Acute pharyngitis | 462 | J02.8,J02.9 |
| Acute tonsillitis | 463 | J03.8,J03.9 |
| Acute laryngitis/tracheitis | 464 | J04,J05 |
| Acute upper respiratory infection of multiple sites/not otherwise specified | 465 | J06 |
| Pneumococcal pneumonia | 481 | J13,J18.1 |
| Other bacterial pneumonia | 482 | A48.1,J14,J15.0-J15.6,J15.8,J15.9 |
| Bronchopneumonia with organism not otherwise specified | 485 | J18.0 |
| Pneumonia, organism not otherwise specified | 486 | J18.8,J18.9 |
| Acute exacerbation of obstructive chronic bronchitis | 491.21 | J44.0,J44.1 |
| Bronchiectasis | 494 | J47 |
| Empyema | 510 | J86.0,J86.9 |
| Lung/mediastinum abscess | 513 | J85 |
| 1. **Cardiovascular system infection** | | |
| Acute pericarditis | 420 | I30 |
| Acute or subacute endocarditis | 421 | I33,I39 |
| Thrombophlebitis | 451 | I80 |
| 1. **Gastrointestinal / Biliary tract system infection** | | |
| Intestinal infection not otherwise classified | 008 | A04,A08 |
| Ill-defined intestinal infection | 009 | A09 |
| Acute appendicitis | 540 | K35 |
| Appendicitis not otherwise specified | 541 | K37 |
| Other appendicitis | 542 | K36 |
| Diverticulitis of small intestine without hemorrhage | 562.01 | K57.00,K57.12 |
| Diverticulitis of small intestine with hemorrhage | 562.03 | K57.01,K57.13 |
| Diverticulitis of colon without hemorrhage | 562.11 | K57.20,K57.32,K57.40,K57.52,K57.92 |
| Diverticulitis of colon with hemorrhage | 562.13 | K57.21,K57.33,K57.81,K57.93 |
| Anal and rectal abscess | 566 | K61 |
| Peritonitis | 567 | K65 |
| Intestinal abscess | 569.5 | K63.0 |
| Perforation of intestine | 569.83 | K63.1 |
| Abscess of liver | 572.0 | K72.1,K72.9,K75.0,K75.1,K76.6-K76.8 |
| Portal pyemia | 572.1 | K75.1 |
| Cholelithiasis | 574 | K80 |
| Acute cholecystitis | 575.0 | K81 |
| Cholangitis | 576.1 |  |
| 1. **Genitourinary system infection** | | |
| Kidney infection | 590 | N10,N11,N12,N13.6,N15.1,N15.9,N16,N28.84-N28.86 |
| Urethritis/ urethral syndrome | 597 | N34.0-N34.3 |
| Urinary tract infection not otherwise specified | 599.0 | N13.9,N36.0-N36.2,N36.41,N36.42,N36.5,N36.8,N36.9,N39.0,N39.8,N39.9,R31 |
| Prostatic inflammation | 601 | N41,N51 |
| Female pelvic inflammation disease | 614 | N70,N73.0-N73.6,N73,N74 |
| Uterine inflammatory disease | 615 | N71 |
| Other female genital inflammation | 616 | N72,N75,N76,N77,N94.810 |
| 1. **Soft tissue/musculoskeletal system infection** | | |
| Erysipelas | 035 | A46 |
| Cellulitis, finger/toe | 681 | L02.51,L02.61,L03.011,L03.0 |
| Other cellulitis or abscess | 682 | K12.2,L02.01,L02.11,L02.21,L03.2,L03.3 |
| Acute lymphadenitis | 683 | L04 |
| Other local skin infection | 686 | B78.1,E83.2,L08.81,L08.82,L08.89,L08.9,L88,L92.8,L98.0 |
| Pyogenic arthritis | 711.0 | M00 |
| Osteomyelitis | 730 | M86 |
| 1. **Device-related infection** | | |
| Infection or inflammation of device/graft | 996.6 | T80.211A,T80.212A,T80.218A,T80.219A,T80.22XA,T82.6XXA,T82.7XXA,T83.51XA,T83.59XA,T83.6XXA,T84.50XA,T84.51XA,T84.52XA,T84.53XA,T84.54XA,T84.59xA,T84.60XA,T85.79XA |
| 1. **Other / undetermined infection** |  |  |
| Cholera | 001 | A00 |
| Typhoid/paratyphoid fever | 002 | A01 |
| Other salmonella infection | 003 | A02 |
| Shigellosis | 004 | A03 |
| Primary tuberculosis infection | 010 | A15.6,A15.7 |
| Pulmonary tuberculosis | 011 | A15.0 |
| Other respiratory tuberculosis | 012 | A15.4-A15.6 |
| Central nervous system tuberculosis | 013 | A17 |
| Intestinal tuberculosis | 014 | A18.3,A18.83 |
| Tuberculosis of bone and joint | 015 | A18.0 |
| Genitourinary tuberculosis | 016 | A18.1 |
| Tuberculosis not otherwise classified | 017 | A18.2,A18.4,A18.5,A18.6,A18.7,A18.81,A18.82,  A18.84,A18.85,A18.89 |
| Miliary tuberculosis | 018 | A19 |
| Plague | 020 | A20 |
| Tularemia | 021 | A21 |
| Anthrax | 022 | A22 |
| Brucellosis | 023 | A23 |
| Glanders | 024 | A24.0 |
| Melioidosis | 025 | A24.1-A24.3,A24.9 |
| Rat-bite fever | 026 | A25 |
| Other bacterial zoonoses | 027 | A26,A28.0,A28.2,A28.8,A28.9,A32 |
| Leprosy | 030 | A30 |
| Other mycobacterial disease | 031 | A31 |
| Diphtheria | 032 | A36 |
| Whooping cough | 033 | A37.0,A37.1,A37.8,A37.90 |
| Streptococcal throat/scarlet fever | 034 | J02.0,J03.0,A38 |
| Meningococcal infection | 036 | A39 |
| Tetanus | 037 | M86 |
| Actinomycotic infections | 039 | A42,A43,B47.1,B47.9,L08.1, |
| Other bacterial diseases | 040 | A48.0,A48.2,A48.3,A48.4,A48.8,K90.81,M60.009 |
| Bacterial infection in other diseases not otherwise specified | 041 | A49,B95,B96, |
| Congenital syphilis | 090 | A50 |
| Early symptomatic syphilis | 091 | A51.0-A51.4 |
| Early syphilis latent | 092 | A51.5,A51.9 |
| Cardiovascular syphilis | 093 | A52.0 |
| Neurosyphilis | 094 | A52.1,A52.2 |
| Other late symptomatic syphilis | 095 | A52.7 |
| Late syphilis latent | 096 | A52.8 |
| Other and unspecified syphilis | 097 | A52.9,A53 |
| Gonococcal infections | 098 | A54 |
| Leptospirosis | 100 | A27 |
| Vincent’s angina | 101 | A69 |
| Yaws | 102 | A66 |
| Pinta | 103 | A67 |
| Other spirochetal infection | 104 | A65,A69.8,A69.9 |
| Dermatophytosis | 110 | B35 |
| Candidiasis | 112 | B37 |
| Coccidioidomycosis | 114 | B38 |
| Histoplasmosis | 115 | B39,G02,I32,I39,J17 |
| Blastomycotic infection | 116 | B40,B41 |
| Other mycoses | 117 | B42,B43,B44.1,B44.2,B44.7,B44.89,B44.9,B45.0,  B45.2,B45.3,B45.7-B45.9,B46,B47.0,B48,B49 |
| Opportunistic mycoses | 118 | B48.8 |
